# Mental Disorder and Toxæmia

**Published:** 1900-12-01

**Authors:** 


### MENTAL DISORDER AND TOXÆMIA.

The theory of the toxæmic origin of disease, although new in name, is as old as the hills, and the idea which it expresses has been at the root of much of the drug treatment of disease for many ages. What seems quite certain is that the normal function of the body depends on the normal environment of its cellular elements, and that in its ultimate analysis this means that the fluid by which every cell is surrounded, and from which it draws its pabulum, is exactly normal in its chemical constitution. If then, as we take to be the case, an excess or a defect in any of the normal elements in the blood may produce disturbance of function, how much more may we expect this result when the cells have to draw their nutriment from a fluid which is charged with foreign and more or less poisonous elements in those conditions which we describe under the general heading of toxæmia!

That the continued feeding upon poisoned pabulum produces disease not only in the more delicate, but even in the coarser tissues of the body no one can doubt who sees the fibrotic changes that sometimes have their origin in this way. Such evidences of injury as are to be found in old fibrosis, and in various degenerative changes, are, however, signs of what has long since gone by rather than of anything happening at the time; and if we are to see in full what effect on function can be produced by poisoned blood, we must watch the results of toxæmia as it affects the most delicate and highly specialised of our tissues, namely, nervous structure; and among nervous structures we must choose for observation those which respond most quickly to altered surroundings and give most unmistakable outward indications of anything wrong in their inner workings. The tissues and the structures then which must take as our most delicate index of the influence of toxæmia upon functions are those which are engaged in mental operations. Much interest thus attaches to the remarks made by Sir Dyce Duckworth in addressing the Medico-Psychological Society last week on "The Mental Disorders dependent on Toxæmia," for we may be quite sure that for every abnormality of cerebral cell activity which makes itself manifest by abnormality of mental state there are corresponding abnormalities of other cell activities all over the body, which, although they do not at once become manifest or show any signs of their occurrence, are none the less real in their influence upon the various bodily functions. If a dose of alcohol, or of some animal toxin can produce changes in brain tissue, the effects of which are at once manifest, we cannot but believe that effects of some sort, hidden though their results may be, are being produced on various cell activities throughout the body. And this thought has two sides, for, while it tends to reduce the causes of disease to a "humoral" or chemical basis, it gives great hope in regard to the possibilities of treatment. If the initial change by which the direction of cell activity is determined be a chemicai one, by chemical means also—that is by drugs—ought it to be possible to set things right. It is in this direction that the researches of physiological chemistry speak hopefully for the future of drug therapeutics. Turning now to the subject of mental disease, Sir Dyce Duckworth says that our first consideration must be to recognise what serious defects may result from minor flaws and limited morbid lesions in certain parts of the brain. It is, he says, astounding to realise that the histologist can in no way distinguish the physical characters pertaining respectively to the brain matter of a philosopher or a cowherd, and yet how vastly different the endowments of each!

The brain, the organ of mind, is only healthy if its thinking parts work in harmonious and physiological order, and this relates essentially to a perfect nutrition and a normal cellular metabolism. There may be a sufficient supply of blood conveyed by healthy arteries, but that supply may be inadequate in quality or deficient in essentials for perfect nutrition.

Again, it may be inappropriate by reason of something positively harmful contained in it. An altered metabolism occurs, and the result is delirium or mental aberration. Were a necropsy possible in such a case there would almost certainly be nothing gross or macroscopical to reveal the nature of it. An ordinary necropsy in cases of acute mania and of melancholia commonly reveals nothing abnormal. The probability is that we are only now gradually arriving at the *vera causa* of various morbid states by the employment of new methods which have relation not so much to the histological condition of the tissues as to the chemical relations which exist between the cell and its environment. We have traced the grosser lesions affecting the brain to the elementary tissues in which they began, but we now require the services of the physiologist and the chemist to discover what are the changes of environment by which these tissue changes are produced.

"Mental health must surely be dependent on the integrity of the brain cells and neurons. Psychic endowment, no less than ordinary nerve energy, requires a normal condition of the machinery for its functions. The nuclei of the myriad cells as physiological centres, their plasmic environment, the processes of the cell, their fibres and their collaterals must be intact, and the subtle metabolism at once securing nutrition and dispensing energy may not be disturbed or perverted without manifest disturbance of psychic and ordinary nervous rhythm."

Sir Dyce Duckworth then went on to describe the various phases of mental disorder which depend on toxæmic states, and said that the origin of the toxins by which they are caused is at least threefold.

They may, he said, be generated within the body (auto-intoxicants), or by the malign working of microbes introduced from without, or by impregnation with poisons of various kinds, including those resulting from habits of alcoholism, chloralism, cocainism, and morphinism. In the majority of cases of insanity probably there is a hereditary predisposition to the disorder, but in some cases a previously normal brain may be so damaged by toxic influences as to manifest aberration, as may be seen in the varied forms of uræmia, in the autotoxy of diabetic coma, or in that which depends on acute atrophy of the liver. The effects of these auto-intoxications are as certain as those of poisons taken by the mouth or hypodermically, and the chemical functions of the brain-cell are disordered in precisely the same manner.

Again, no clearer evidence of auto-intoxication could be afforded than that which results in aberrant cerebration in some cases of gout. The patient generates his own toxins in his tissues, and the effects are recognised in the form of stupor, delirium with delusion and excitement, sometimes as melancholia, such symptoms lasting for days or for many weeks, and yielding to some overt metastatic gouty development, generally articular, or to an anti-gouty medication, with complete recovery.

As the result of direct toxic infections we may have various grades of mental aberration in enteric, typhus, and rheumatic fevers, erysipelas, cholera, influenza, diphtheria, and scarlet fever. The malign cerebral distempers due to influenza are very definite. All forms of insanity may result from it, and although there is reason to believe that in most instances there may have been an underlying neurotic proclivity, it is certain that the specific influenzal toxin affects the nervous system very gravely in most cases. According to Dr. John Taylor all forms of acute mania are probably of toxic origin, affecting wide areas of brain tissue.

Finally we have the toxæmic states induced by impregnation with certain organic poisons. Lead stands first among these, not only having in itself a directly injurious effect upon nerve tissue, but having also such an influence on general metabolism as to lead to the production of various auto-toxins. Lastly, there are the mental aberrations due to the vicious use of such agents as morphine, chloral hydrate, paraldehyde, ether, and cocaine. There can be no doubt that Sir Dyce Duckworth's address presented in a striking manner the wide influence of toxic agents in producing mental disorders, and in speaking to psychologists that perhaps was the end of the matter. But to the general physician it is only the beginning, for if these mental aberrations are to be taken as showing how greatly function may be disturbed by slight chemical changes in the environment of cell life we seem to have the key to many morbid changes in other parts of the body—changes which from the very fact that they show no sign at the time are enabled to continue and progress until definite organic changes are produced.

